# Laws on filial support in four Asian countries

**DOI:** 10.2471/BLT.17.200428

**Published:** 2017-10-02

**Authors:** Ray Serrano, Richard Saltman, Ming-Jui Yeh

**Affiliations:** a Department of Health Policy and Management, Rollins School of Public Health, Emory University, 1518 Clifton Road NE, Atlanta, GA 30322, United States of America.

Throughout the world, national policy-makers are being pushed, by ageing populations, low fertility rates and changing cultural values, to find innovative and sustainable policy solutions to meet the needs of older people. Since the mid-1990s, policy-makers in Bangladesh, China, India and Singapore have developed filial-support laws as a way of meeting some of these needs. Although such laws are not panaceas for addressing all of the problems associated with sociodemographic change, in resource-limited settings, they can help persuade families to provide a greater share of the social and health-care needs of their old members.

Filial-support laws create “a statutory duty for adult children to financially support their parents who are unable to provide for themselves.”^1^ The laws assign filial responsibilities and establish the extent to which adult children must provide for their ageing parents. The laws are predicated on two conditions. First, governments must have a systematic mechanism for determining need. Many filial-support laws stem from social-welfare laws and identify a person in need as an individual who is “so poor that they must be supported at public expense.”^1^ Second, the statutes themselves must assign responsibilities based on the traditional concepts of intergenerational fairness and reciprocity. For example, the common belief that, in adulthood, children should compensate their parents for the sacrifices that their parents made in supporting them to adulthood. Adult children could be relieved of their filial responsibilities if their parents were known to have abandoned their parental obligations at some point during their children’s upbringing. In such cases, family courts or special tribunals can act as adjudicators and or arbitrators.

For centuries, many Asian communities have benefitted from deep-seated views of filial piety and respect for elders.^2^ These views have created such strong family-support mechanisms that some national governments have, apparently, never felt the need to maintain welfare programmes for old people. In some Asian countries, however, the efficiency and sustainability of the family-support mechanisms have been challenged in recent decades by large increases in life expectancy and in the prevalence of chronic disease. In response, the governments of countries such as Japan and Singapore have enacted policies that expand social and financial services, such as welfare-state services for old people.^2^

## Filial-support laws in Asia

Table 1 highlights the variance among the filial-support laws of four Asian countries: Bangladesh, China, India and Singapore. Singapore’s Maintenance of Parents Act of 1995 details the monthly allowance or lump sum to be paid by adult children for the maintenance of their parents.^7^ Although this law does not specify a penalty for noncompliance, it did create a mechanism by which individuals older than 60 years can file claims against their children for not providing for their care and well-being.^7^ In India, the Maintenance and Welfare of Parents and Senior Citizens Act of 2007 was an attempt to support the familial care of all old people, including those with no surviving children, and to stipulate civil and criminal penalties for noncompliance.^8^ The act also established a tribunal not only to review claims but also to file claims on behalf of old people.^8^ Both Bangladesh and China amended previous family codes to include a specific requirement that adult children provide for the needs of their parents. Although China’s Law for the Protection of the Rights and Interests of the Elderly of 2013 does not specify a penalty, it strongly encourages adults to consider the health-care and social needs of their older relatives.^3^ In Bangladesh, the Parents Maintenance Act of 2013 specifies that noncompliance should lead to fines and, if the fines go unpaid, a period of incarceration.^9^

**Table 1:** Filial-support laws, Bangladesh, China, India and Singapore, 1995–2013

Country	Name of law	Year enacted	Main requirements	Can third-parties file under the act?	Penalties	No. of cases
Bangladesh	Parents Maintenance Act	2013	If adult children do not provide their parents’ maintenance without any “logical reason”, the parents may get remedy by complaint. All adult children must provide their parents with a “logical amount of money” for maintenance, from their earnings, if the parents do not live with the children. In the absence of one or both parents, the grandparents may be entitled to “maintenance” allowances.	NS	Fines of up to US$ 1200. If fines are unpaid, violators may face imprisonment for up to 3 months.	NA^a^
China	Law for the Protection of the Rights and Interests of the Elderly	2013	“Family members should care for the spiritual needs of the elderly and must not ignore or neglect them. The supporters who live separately from the elderly should frequently visit or send a greeting”^3^	Article 43 notes: “When the lawful rights and interest of the elderly are infringed upon, they or their agents shall have the right to refer the matter to the department concerned or bring a lawsuit to a people's court according to law”^3^	NS	In Beijing, 63 tribunals in 2016 and 290 between 2013 and 2017. Corresponding numbers for China were 2754 and 8647, respectively^4^^,b^
India	Maintenance and Welfare of Parents and Senior Citizens Act	2007	Adult children and or grandchildren are under obligation to maintain at least one parent or grandparent. An adult relative of a senior citizen is bound to look after the senior citizen.	Yes, but only a tribunal may file complaints on behalf of old people	Fines of US$ 75 and or imprisonment for up to 3 months	In the National Capital Territory of Delhi, 571 tribunals in 2017 and 1651 between 2009 and 2017^5^^,c^
Singapore	Maintenance of Parents Act	1995	Adult children must pay each Singaporean parent who is aged at least 60 years, either a monthly allowance, or a lump sum, for maintenance	The act does not specify if third parties can file complaints on behalf of old people	NS	There were 70 new applicants for maintenance in 2016 and 2283 between 1996 and 2016^6^

NA: not available; NS: not specified; US$: United States dollars.

^a^ Data on the numbers of cases do not appear on the web sites of the Bureau of Statistics or the Ministry of Social Welfare.

^b^ China’s national government does not publicly post data on the numbers of cases. The numbers shown here are crude estimates (probably underestimates) based on the results of a search of the online databases of the supreme people's court for which “Law for the Protection of the Rights and Interests of the Elderly” and “maintenance” were used as search terms.^4^

^c^ In India, the decentralized enforcement of the act means that no national statistics on the numbers of cases are available.^5^

## Economic and social benefits

In general, filial-support laws supplement current government efforts to support old people. In recent decades, countries such as Bangladesh and India have steadily increased means-tested cash payments to individuals aged at least 60 years.^1^ In spite of such increases, however, such payments remain insufficient to meet the basic needs of old people and additional support from families is often needed.

Compared with the number of old people in China, India and Singapore, the national numbers of cases or appeals relating to the filial-support laws appear quite small (Table 1). However, as such cases attract a lot of media attention, they may improve compliance nationwide and motivate families to increase their support of old relatives. Moreover, filial-support laws can complement intra-family discussions on retirement planning and asset transfers. Such benefits are evident in Singapore, where family-support mechanisms remain strong in spite of demographic changes and limited social-welfare programmes. For example, well over 70% of the respondents involved in Singapore’s 2011 National Survey of Senior Citizens, reported that cash transfers from children represented their greatest source of income.^10^ Since 1995, when Singapore enacted its filial-support law, old Singaporeans have taken on increasing responsibility for their own maintenance. For example, an increasing proportion of this age group is supplementing any asset transfers from their children by remaining employed after an age of 60 years.^10^

Compared with high-income Singapore, retirement planning in low- or middle-income Bangladesh, China and India seems more daunting, because of weak or non-existent pension schemes. In Bangladesh, for example, the agriculture sector, migrants and those in self-employment are not covered by the pension schemes currently afforded to public-sector employees.^7^ Most old people in Bangladesh and particularly those living beyond the official retirement age of 57 years have to rely on family for their maintenance.^7^ Low economic growth, poverty, and increasing dependency ratios have all had negative impacts on the traditional systems of family support for old Bangladeshis. There may have to be compensations, in terms of new state initiatives and institutional arrangements, to ensure sustainable care and well-being. In China, rapid economic growth led to a massive rural-to-urban migration that has left nearly half of those aged at least 60 years, living well apart from their children.^2^ While China’s social-welfare system is deepening its packages of services, there remains a steady breakdown in traditional family structures that has left many old Chinese, particularly the old and poor, fending for themselves. In India, although about three-quarters of those aged at least 60 years still co-reside with their spouses, children and grandchildren, the proportion of this age group that either lives alone or only with a spouse increased from 9% in 1992 to 19% in 2006.^11^

## Implementation challenges

Given the general paucity of social-welfare programmes, the well-being of old people in low-and middle-income countries such as Bangladesh, China and India may not be fully protected by legislation that emphasizes filial responsibility. One challenge is the full and fair enforcement of filial-support laws, especially when the relevant information, for example, on the place of residence, employment and income of adult children, may be hard to obtain. Another dimension to examine is the general respect for laws in any given country. According to the World Bank, there is wide between-country variance in the extent to which residents have confidence in, and abide by, national legislation.^12^ Public perceptions of the quality of contract enforcement, property rights, the police and the courts are important, if not determinant of the success of filial-support and other laws.

A major social problem stemming from filial-support laws is the possibility that enacting such laws may diminish gains in gender equality. Across low- and middle-income countries, women provide about 70% of the physical care for old people and wives provide most of the physical care, not only for their own parents, but also their parents-in-law.^1^ There is a risk that, as families become increasingly legally responsible for the care of old relatives, employed women may resign to become unpaid caregivers for old people.

Finally, it remains unclear whether many old people with children who do not comply with filial-support laws would sue their children. By doing so, this would acknowledge that the unofficial intergenerational contract has failed and that their children have violated civil and or criminal codes.

## Conclusion

The direct and indirect effects of the filial-support laws enacted in Asia since 1995 merit further research. In low- and middle-income countries, additional resources may be needed to provide for the care and well-being of old people. It remains unclear if, ultimately, filial-support laws that appear to work well in high-income countries, such as Singapore, can persuade families in more resource-limited settings to address the needs of their older relatives. In all settings, compared with national governments, family members may be better at assessing the needs of old people and may be able to respond more appropriately and more quickly to such needs. While governance mechanisms may be weak in particular countries, filial-support laws may still help to stimulate voluntary compliance by families. With effective enforcement, compliance, particularly among the wealthy and better educated, may rise. Increasing compliance could free up services that can then reach old people with inadequate or no familial support.
